# Acoustoelectric Effect of Rayleigh and Sezawa Waves in ZnO/Fused Silica Produced by an Inhomogeneous In-Depth Electrical Conductivity Profile

**DOI:** 10.3390/s23062988

**Published:** 2023-03-09

**Authors:** Cinzia Caliendo

**Affiliations:** Institute for Photonics and Nanotechnology, IFN-CNR, Via del Fosso del Cavaliere 100, 00133 Rome, Italy; cinzia.caliendo@cnr.it

**Keywords:** acoustoelectric effect, UV, ZnO, Rayleigh wave, Sezawa wave

## Abstract

The acousto-electric (AE) effect associated with the propagation of Rayleigh and Sezawa surface acoustic waves (SAWs) in ZnO/fused silica was theoretically investigated under the hypothesis that the electrical conductivity of the piezoelectric layer has an exponentially decaying profile akin to the photoconductivity effect induced by ultra-violet illumination in wide-band-gap photoconducting ZnO. The calculated waves’ velocity and attenuation shift vs. ZnO conductivity curves have the form of a *double*-relaxation response, as opposed to a *single*-relaxation response which characterizes the AE effect due to surface conductivity changes. Two configurations were studied which reproduced the effect of UV light illumination from the top or from the bottom side of the ZnO/fused silica substrate: 1. the ZnO conductivity inhomogeneity starts from the free surface of the layer and decreases exponentially in depth; 2. the conductivity inhomogeneity starts from the lower surface of the ZnO layer contacting the fused silica substrate. To the author’s knowledge, this is the first time the double-relaxation AE effect has been theoretically studied in bi-layered structures.

## 1. Introduction

Zinc oxide (ZnO) is a wide bandgap (∼3.4 eV) II-–VI compound with excellent piezoelectric properties. If doped with properly selected transition metals, such as Mn, the ZnO optical absorption spectrum extends from the UV to the visible region; moreover, Ag-decorated Mn:ZnO nanocomposites can be applied in high-efficiency photocatalysis [1]. ZnO can be grown in thin film form by means of different techniques including reactive sputtering, or laser molecular beam epitaxy or pulsed laser deposition, etc. [2,3,4], thus allowing the transduction of surface acoustic waves (SAWs) onto non-piezoelectric substrates, such as SiO_2_, by means of metal interdigitated electrodes (IDTs) patterned onto the piezoelectric layer surface. By applying a RF signal to the IDT, the piezoelectric material is excited to generate propagating SAWs whose characteristics (such as frequency, number of acoustic modes, amplitude, and wave-front orientation) are defined by the geometrical dimensions of the electrodes and the layer thickness.

Fundamental and higher-order surface wave modes can propagate in a single ZnO/SiO_2_ device structure depending on the value of the ZnO layer thickness-to-wavelength ratio, h/λ: these modes are called Rayleigh, Sezawa, and higher-order Rayleigh waves. These modes are characterized by different velocities and thus resonant frequencies. When the SAWs propagate in a piezoelectric medium, both mechanical and electric potential fields travel synchronously: as a result, the waves’ propagation characteristics (phase velocity and propagation loss) can be perturbed if the electrical boundary conditions are changed as this happens in the presence of a thin conducting layer covering the surface of the piezoelectric substrate. As a result, the charge carriers in the film redistribute and generate an electrical field to compensate for the electrical field of the bound charges; the interaction of the free carriers with the electric field accompanying the propagating SAW in the piezoelectric material causes a reduction in the phase velocity and an increase in the propagation loss of the wave. This is the *surface* AE effect as opposed to the *volume* AE effect which takes place when the piezoelectric material undergoes a non-uniform conductivity change across its depth due to illumination by ultraviolet (UV) light at 365 nm, which corresponds to the photon energy equal to the ZnO band gap; free carriers are photogenerated, resulting in a non-uniform conductivity change across its depth [5]. Since the ZnO recovers the double role of the UV-sensitive layer and SAWs transducer, a careful study of the device’s performance is required as a function of some design parameters, such as the UV-sensitive layer thickness, the acoustic wavelength, the electrical boundary conditions, the UV penetration depth, the non-piezoelectric substrate material type, and crystallographic orientation, to cite just a few.

In the present paper, the AE effect associated with the propagation of the Rayleigh and Sezawa modes in ZnO/fused silica is theoretically studied under the hypothesis that the ZnO layer has an exponentially decaying conductivity profile.

The advantage of using a multi-frequency operation in a single device structure (ZnO/fused silica) is the possibility of analyzing multiple acoustic modes (Rayleigh and Sezawa modes with different excitation frequencies) that are each reacting differently on the external stimulus (the UV radiation adsorption). Rayleigh and Sezawa modes are excited by the same IDTs (with metal fingers with a fixed periodicity, λ) placed onto the same substrate: as the two modes travel at different phase velocities, they have different resonant frequencies. Moreover, the ZnO/SiO_2_-based device can be driven on the harmonic modes of the two waves, yielding higher-sensitivity sensors.

The two modes are characterized by different electric potential distribution between the surface and the bulk of the propagating medium; thus, they can show different sensitivity to electrical perturbations. Depending on the ZnO thickness-to-wavelength ratio h/λ, even higher-order Rayleigh waves can be excited in a single device structure; in a multi-mode setup, the parallel readout of several frequencies (with different sensing properties) offers the possibility to perform measurements with increased accuracy.

The advantage of using a transparent silica substrate in combination with the ZnO sensing layer is that the bi-layer can be illuminated by the UV light from the back (through the fused silica substrate which has a window of high transmission extending from the deep ultraviolet to the infrared region [6]) or from the top. Finite element simulations were performed which revealed that the two different sensing configurations react differently to the UV exposure and that this difference depends on the SAW mode.

The purpose of this paper is to study the AE effect of SAW interacting with in-depth inhomogeneous conductivity profiles, which is akin to the photoconductivity effect induced by ultra-violet illumination in wide-band-gap photoconducting ZnO. The calculated waves’ velocity and attenuation shift vs. ZnO conductivity curves have the form of a *double*-relaxation response, as opposed to a *single*-relaxation response which characterizes the AE effect based on surface conductivity changes. Since the UV penetration depth in ZnO depends on the optical wavelength of the UV band, the sensor responses were calculated for some skin depth values in the range from 100 to 500 nm.

The paper is organized as follows: the propagation characteristics (phase velocity and electroacoustic coupling coefficient dispersion curves) of Rayleigh and Sezawa modes in ZnO/fused silica substrates are described in Section 2; the AE effect response (wave velocity and propagation loss shifts) to conductivity changes on the surface of piezoelectric substrates is described in Section 3 and is in agreement with the perturbation theory prediction; the simulation methodology for investigating the AE effect induced by inhomogeneous conductivity changes in ZnO/fused silica structures is described, and the theoretical results are shown, in Section 4; the theoretical results are discussed in Section 5; the concluding paragraph shows a table listing some experimental results published in the available literature on the performances of ZnO-based UV SAW sensors and outlines some possible future trends. To the best of the author’s knowledge, the double-relaxation AE effect has not yet been observed experimentally or studied theoretically, except for reference [7] where it is theoretically studied under Rayleigh waves propagation in piezoelectric ZnO half-spaces.

## 2. Surface Acoustic Waves Propagation along ZnO/SiO_2_

Rayleigh waves are surface acoustic waves (SAWs) that propagate through the surface of piezoelectric substrates, and their amplitude decreases exponentially with the depth. Piezoelectric materials are characterized by the following feature: they can generate an electric potential when subjected to an external mechanical strain or a mechanical strain under an external electric field. As a result, the Rayleigh waves are characterized by three particle displacement components and an electrical potential wave, Φ, moving in synchrony; the displacement components and electric potential are given by the following equations:

(1) $$u_{i}=U_{i}^{0}e^{jkbz}e^{jk\left(x-vt\right)}$$

(2) $$\Phi =\Phi_{0}e^{jkbz}e^{jk\left(x-vt\right)}$$

in which *k* is the wave number, $j=\sqrt{-1}$, *x* is the wave propagation direction, *z* is the vertical direction, *v* and *t* are the velocity and time, respectively, $U_{i}^{0}$ (for *i* = 1, 2, 3) and Φ_0_ are the amplitudes of the particle displacements and electric potential, and *kb* is the wave number along the direction perpendicular to the surface. The SAWs travelling along the surface of the half-space (*z* = 0 is the surface and the *z* axis points toward the bulk) must satisfy mechanical and electrical boundary conditions: 1. the normal components of the stress tensor, $T_{3i}=0$, must be zero at the free surface (*z* = 0); 2. $U_{i}$ and Φ must vanish at large depths (*z* → ∞); 3. Φ and the normal component of the electric displacement must be continuous across the free surface (*z* = 0) of the piezoelectric half-space. Detailed theoretical aspects of acoustic wave propagation in solid media are discussed in references [8,9].

**Figure 1:**
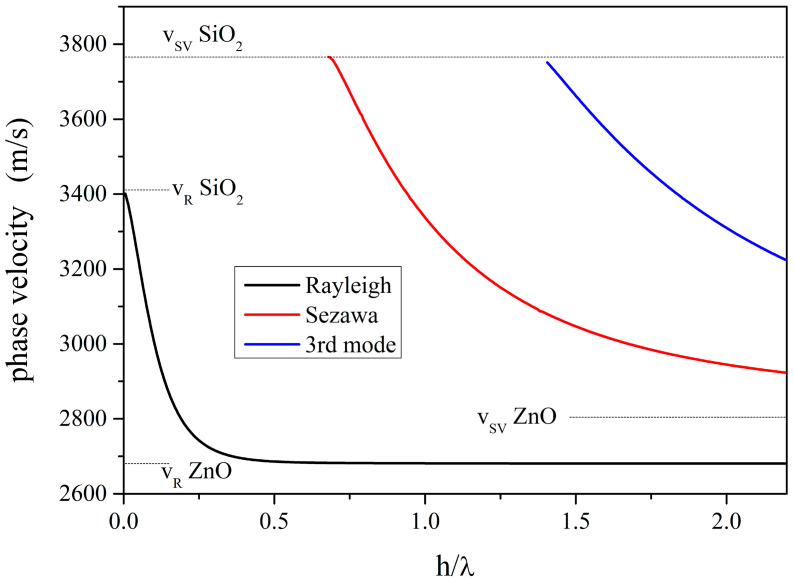
The phase velocity dispersion curves of the Rayleigh waves in the multi-mode SiO_2_/ZnO structure.

The SAW velocity depends on the crystallographic cut of the propagating medium and on the wave propagation direction [8,9]; the c-ZnO is isotropic in the c-plane, and thus, the SAW velocity does not depend on the wave propagation direction. In single-material half-space, the confinement mechanism of the SAWs depends on the presence of a stress-free surface. If the propagating medium consists of a layer/substrate structure, it can sustain the propagation of multiple SAWs, named Rayleigh, Sezawa, and higher-order Rayleigh modes: the condition required for this to happen is that the transverse bulk wave velocity of the half-space is larger than that of the layer material. The ZnO/fused silica structure is an example of a medium which sustains the propagation of multiple SAW modes since the transverse bulk wave velocity of the fused silica (3766 m/s) is larger than that of the ZnO (2806 m/s). The number of SAW modes and their velocities depend on the layer thickness, h: with a very thin film thickness (h/λ << 1), only the fundamental Rayleigh mode propagates with a velocity very close to the SAW velocity of the substrate material (3411 m/s); by increasing the layer thickness (h/λ >> 1), the Rayleigh velocity asymptotically reaches the SAW velocity of the layer material (2644 m/s). The second order Rayleigh mode is generally called the Sezawa mode, and the other modes are simply called R3, R4, and so on. The amplitude profile of the Rayleigh wave is predominantly confined in the layer and decays exponentially with the depth, while that of the higher order modes has an exponential tail in the substrate. The latter modes have a layer thickness-to-wavelength cut-off at which the phase velocity is equal to the substrate shear velocity. Right at the cut-off, the SAW mode couples with bulk modes and shows a leaky nature, as the acoustic power flows into the bulk substrate, thus resulting in a large insertion loss. By increasing the layer thickness, the velocity of the higher order modes asymptotically reaches the shear velocity of the layer [10]. Figure 1 shows the Rayleigh, Sezawa, and third Rayleigh mode phase velocity dispersion curves vs. the ZnO thickness-to-wavelength ratio, h/λ. The data shown in Figure 1 were numerically calculated by using the McGill software [11]. The surface of the ZnO layer was assumed electrically and mechanically free, and all the materials were assumed lossless. The velocity of the shear bulk wave (v_SV_ = 3766 m/s) and of the Rayleigh wave (v_R_ = 3411 m/s) in the quartz substrate are shown in Figure 1 as well as the velocity of the Rayleigh wave and of the shear bulk wave in ZnO (v_R_ = 2644 and v_SV_ = 2806 m/s). The velocity of the Rayleigh wave (black curve) is equal to the SAW velocity in the quartz substrate under h/λ = 0 (bare substrate) and decreases up to the SAW velocity in ZnO with increasing h/λ. The velocity of the Sezawa wave (red curve) is equal to the velocity of the shear bulk wave in the quartz substrate under h/λ equal to the Sezawa cut-off (h/λ = 0.61); with increasing ZnO h/λ, the velocity asymptotically reaches the velocity of the bulk shear wave in ZnO.

With very thin film thicknesses (h/λ up to about 0.61), only the Rayleigh mode propagates; the Sezawa mode appears as it exceeds its cut-off value (h/λ > 0.61). With further increases in h/λ, the third and higher order Rayleigh modes appear whose velocities asymptotically reach the shear bulk wave velocity in ZnO. Depending on the ZnO h/λ, one mode or more than one mode can propagate in the ZnO/SiO_2_ substrate.

**Figure 2:**
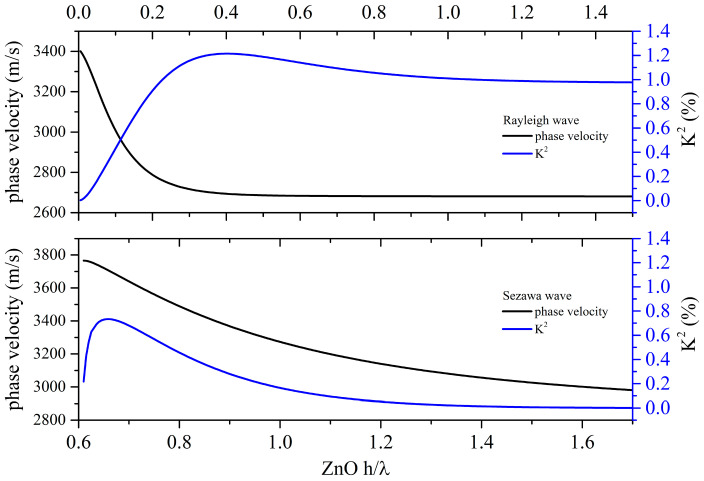
The phase velocity and *K*^2^ dispersion curves of the Rayleigh and Sezawa waves in ZnO/fused silica.

Piezoelectric materials is characterized by a parameter named “electroacoustic coupling coefficient” *K*^2^ which is proportional to the electric-to-acoustic energy conversion efficiency by electrical means, the IDTs. *K*^2^ is expressed as twice the relative velocity change due to the electrically free and shorted boundary condition at the surface of the piezoelectric propagating medium, as follows: $K^{2}=2\frac{v_{free}-v_{met}}{v_{free}}$, $v_{free}$ and $v_{met}$ being the wave velocity of the electrically opened and shorted free surface. For SAW travelling along the surface of a piezoelectric half-space, the *K*^2^ has a single value which depends on the crystallographic orientation of the propagating medium. When the SAW travels along the surface of a double-material substrate, as in the case of ZnO/fused silica, the *K*^2^ becomes dispersive as it depends on the layer thickness as well as on the substrate and layer characteristics. Figure 2 shows the phase velocity and *K*^2^ dispersion curves for the Rayleigh and Sezawa modes propagating along the bi-layered structure ZnO/fused quartz.

## 3. The AE Effect

The propagation of a SAW in a piezoelectric half-space is associated with an oscillating electric field at the sound wavelength which provides an additional elastic stiffness. As a result, a layer of bound charges at the surface of the piezoelectric propagating medium is formed: if the surface electrical conductivity of the medium is perturbed (for example by placing a conductive thin film along the SAW path), the charge carriers in the film redistribute and generate an electrical field to compensate for the electrical field of the bound charges; as a result, the SAW velocity decreases and the SAW propagation loss increases. This phenomenon is called the acousto-electric effect (AE) and is induced by electrical conductivity changes that take place at the surface of the piezoelectric half-space. The AE effect can also be induced by the absorption of gas molecules onto the surface of a thin sensing layer covering the acoustic wave path: the adsorption of an oxidizing or reducing gas changes the electrical conductivity of the sensitive layer [12] according to the number of gas molecules adsorbed. The SAW velocity and attenuation changes represent the response of the SAW gas sensor. According to the perturbation theory [13,14], the SAW velocity and attenuation changes are directly related to the *K*^2^ of the piezoelectric half-space according to the following approximate formulas [13,14]:

(3) $$\frac{\Delta v}{v_{0}}=-\frac{K^{2}}{2}\frac{\sigma_{s}^{2}}{\sigma_{s}^{2}+{\left[v_{0}\left(\varepsilon_{0}+\varepsilon_{s}\right)\right]}^{2}}$$

(4) $$\frac{\Delta \alpha }{k}=\frac{K^{2}}{2}\frac{v_{0}\left(\varepsilon_{0}+\varepsilon_{s}\right)\cdot \sigma_{s}}{\sigma_{s}^{2}+{\left[v_{0}\left(\varepsilon_{0}+\varepsilon_{s}\right)\right]}^{2}}$$

Δα being the loss change, *σ_s_* = *σ* · *h* is the sheet conductivity of the layer, *h* and σ are the conductive layer thickness and bulk conductivity, *ε*_0_ and *ε_s_* are the dielectric permittivity of air and of the piezoelectric half-space, *k* = 2π/λ is the wavenumber, $\Delta v=v-v_{0}$, $v_{0}$ is the unperturbed SAW velocity (the velocity of the SAW travelling along the bare surface of the piezoelectric half-space), v is the SAW velocity perturbed by the presence of a layer with variable conductivity, and σ_c_ = $v_{0}\left(\varepsilon_{0}+\varepsilon_{s}\right)\;$ is the critical conductivity corresponding to the attenuation peak. The maximum value of the relative velocity change, Δvv0, is equal to *K*^2^/2, and the maximum acoustoelectric attenuation is *K*^2^/4. The larger the *K*^2^, the greater the SAW response to the conductivity change on or near the SAW device surface [7]. The theoretical background of the derivation of Formulas (1) and (2) can be found in [13,14].

### Simulation Methodology and Results

**Figure 3:**
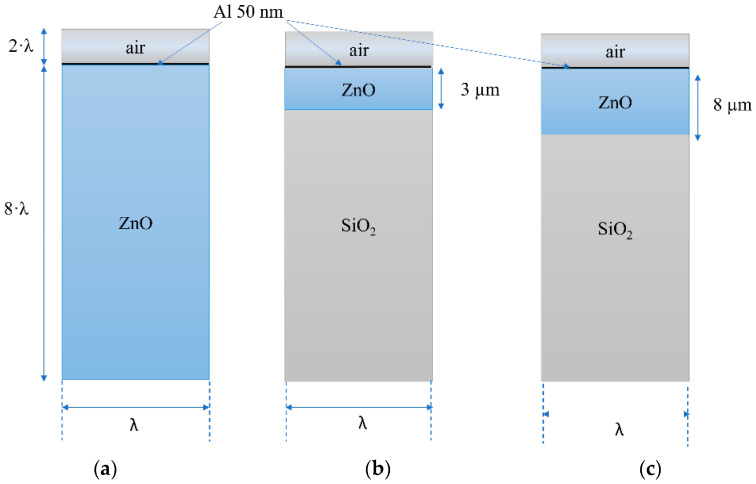
The unit cell made of (**a**) air/Al (50 nm)/ZnO half-space; (**b**) air/Al (50 nm)/ZnO (3 μm)/SiO_2_ half-space; (**c**) air/Al (50 nm)/ZnO (8 μm)/SiO_2_ half-space. The picture is not in scale.

Since the AE effect is driven by the *K*^2^ of the structure, according to the perturbation theory, three different ZnO-based configurations were studied which correspond to three different *K*^2^ values: 0.98 % for the Rayleigh wave propagating in the ZnO half-space, 1.2% for Rayleigh wave in ZnO (h/λ = 0.3)/SiO_2_, and 0.89 % and 1.096% for Sezawa and Rayleigh waves in ZnO (h/λ = 0.8)/SiO_2_, respectively. For h/λ = 0.3, only the Rayleigh wave propagation is excited, while for h/λ = 0.8, two modes propagate. A 2D FEM study was performed by the Comsol Multiphysics 5.6 software to evaluate the SAW velocity changes both in the ZnO half-space and in the ZnO/SiO_2_ substrate due to the ZnO surface conductivity changes induced by the presence of a thin Al layer. The model uses a piezoelectric multiphysics coupling node with the Solid Mechanics and Electrostatics interfaces. The 2-dimensional study is allowed since SiO_2_ is an isotropic material and the c-ZnO is isotropic in the c plane. The unit cell, shown in Figure 3a–c, had a width equal to one wavelength (λ = 10 μm) and consisted of four domains representing the SiO_2_ substrate (8 λ thick), the layer of ZnO (3 or 8 μm thick), the Al layer (50 nm thick), and the air domain (2·λ thick). Both mechanic and electrical fields were considered for the SiO_2_, ZnO, and Al layer (only electrical field for the air domain); the periodic boundary condition was applied to the left and right boundaries of the unit cell so reflections caused by the free edges could be ignored.

An eigenfrequency study was performed to calculate the Rayleigh and Sezawa resonant frequencies, *f*_0_, by using extremely fine meshes (automatically generated physics-defined triangular elements). A sweep parameter study was performed to calculate the mode eigenfrequency for different Al electrical conductivities, σ. It was assumed that the Al thin layer has a complex permittivity: its imaginary part is frequency-dependent according to *j*·σωε0, where σ is the sweep parameter and ω=2·π·*f*_0_. As a result, the resonant frequency, *f_r_*, of the SAW (which is related to the physical constants of all the materials constituting the propagation medium) becomes complex: the real part of *f_r_* is related to the wave velocity, *v*, through the expression Real(*f_r_*) = *v*/*λ*; the imaginary part of *f_r_* accounts for the wave energy losses. 

**Figure 4:**
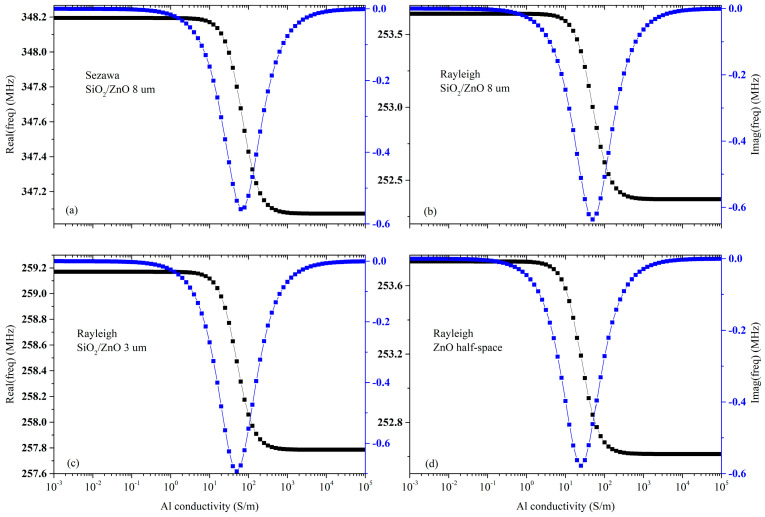
The real and imaginary part of the resonant frequency vs. Al conductivity of the (**a**) Sezawa wave travelling along Al/ZnO (8 μm)/SiO_2_ substrate; (**b**) Rayleigh wave travelling along the Al/ZnO (8 μm)/SiO_2_ substrate; (**c**) Rayleigh wave travelling along Al/ZnO (3 μm)/SiO_2_ substrate; (**d**) Rayleigh wave travelling along a ZnO half-space covered by the thin Al layer. The acoustic wavelength is λ = 10 um and the Al layer is 0.05 um thick.

Figure 4a–d shows the real and imaginary parts of the resonant frequency vs. Al conductivity curves for the three configurations of Figure 3. 

**Figure 5:**
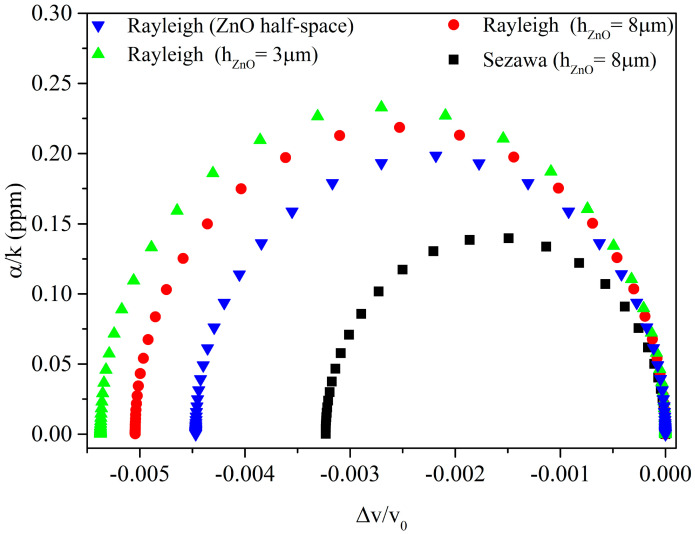
The *α*/*k* vs. the relative phase velocity curves for the Rayleigh wave in ZnO half-space (blue dots), SiO_2_/ZnO (green and red dots for ZnO layer 3 and 8 μm thick), and for the Sezawa wave (black dots) in SiO_2_/ZnO (8 μm), the Al conductivity being the parameter.

As an example, Figure 5 shows the relative phase velocity shift, Δvv0, vs. the *α*/*k* curves, for the Rayleigh and Sezawa waves in the three configurations of Figure 3, *k* being the wavevector, *α* being the propagation loss of the wave, α/k=−54.6·Imagfk·Realf, and the Al conductivity being the parameter.

The Sezawa wave in ZnO/SiO_2_ (black dots) has a *K*^2^ (0.89%) lower than that of the Rayleigh wave (blue dots) in the ZnO half-space (*K*^2^ = 0.98%); the Rayleigh wave in ZnO (3 and 8 μm)/SiO_2_ (green and red dots) has the largest *K*^2^ (1.2 and 1.096%). From Figure 5, it is evident that the magnitude of the AE response of the Rayleigh and Sezawa waves in ZnO/SiO_2_ is related to the corresponding *K*^2^ values. The *surface* AE response has the same shape under the two wave types (Rayleigh and Sezawa waves) as well as under the two types of propagating medium (ZnO half-space and ZnO/SiO_2_ bilayer); differences can be noted in the σ range where the AE response takes place: the range is from 1 to 100 S/m under the Rayleigh wave in ZnO half-space, and from 10 to about 150 S/m under both the Rayleigh and Sezawa waves in the bilayers. Due to the dispersive behavior of the *K*^2^ of the ZnO/SiO_2_ structure, h/λ values can be selected which ensure a high *K*^2^, and hence a conductometric sensitivity fairly larger than that of the ZnO half-space, both in terms of frequency and IL changes per unit of conductivity change.

## 4. The Volume AE Effect

The calculations previously performed are referred to as the surface conductivity change of the piezoelectric medium, which results in the *surface* AE effect. If the phenomenon producing the changes in the electrical conductivity of the piezoelectric medium is, for example, the absorbance of UV radiation, then the UV-induced AE effect can be viewed as a volume effect, hereafter named *volume* AE effect. The UV radiation penetrates the photosensitive piezoelectric medium (which can be a half-space or a layer covering a substrate) and decays exponentially into the depth. It is assumed that the exponential law of light adsorption into the ZnO is equal to the law that defines the spatial distribution of the ZnO photoconductivity features, σzσ0=e−z/δUV, where *δ*_UV_ is the absorption depth, the distance into the material at which the light drops by a factor of 1/e. The method adopted to account for the inhomogeneous conductivity of the ZnO under UV light illumination was to discretize the conductivity depth variation in the ZnO layer and to consider it as a stratified material with characteristics slowly varying over the layers [7].

**Figure 6:**
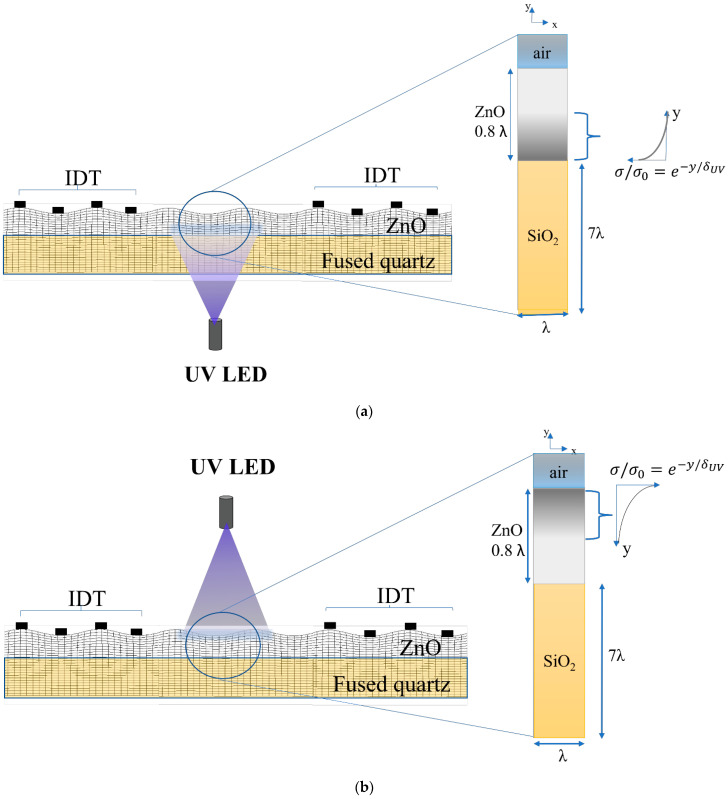
The schematic diagram of the ZnO/SiO_2_-based device under UV illumination (**a**) from top and (**b**) from bottom. The shaded gray scales in the details represent the conductivity profiles (induced by the UV adsorption) inside the ZnO layer. The picture is not in scale.

Two different UV lighting conditions were considered: 1. top lighting, when the source of UV light is placed above the ZnO/fused silica; 2. bottom lighting, when the optical source is below the ZnO/fused silica. As a result, the conductivity profile of the ZnO layer is directed upwards under bottom illumination and downward under top illumination as shown in Figure 6a,b, where the colored gray scale represents the photoconductivity profile inside the ZnO layer under top and bottom illumination.

### Simulation Methodology and Results

**Figure 7:**
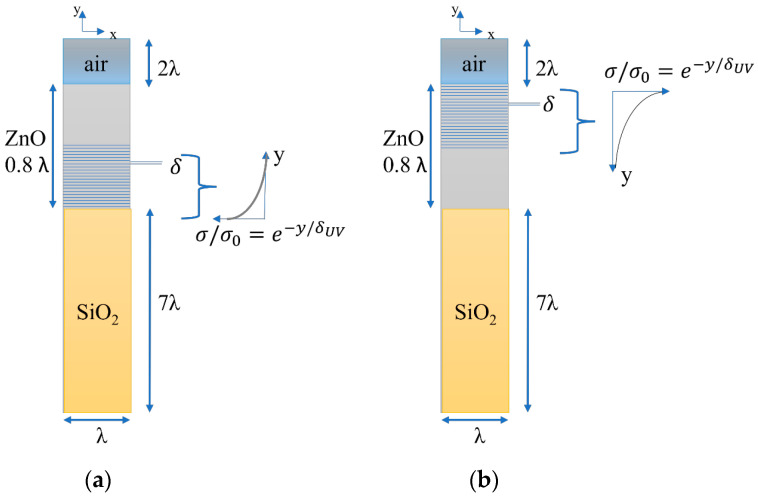
The ZnO/SiO_2_-based unit cell under UV illumination (**a**) from bottom and (**b**) from top. The picture is not in scale.

The propagation of Rayleigh and Sezawa waves in ZnO/SiO_2_ was studied for λ = 10 μm and a ZnO thickness of 8 μm (h/λ = 0.8). The method adopted to account for the inhomogeneous conductivity of the ZnO was to consider it as a stratified material discretized in 100 layers (each with a thickness of *δ* = 30 nm) and one 5 μm thick sub-layer: the latter has a real and fixed permittivity, while the former have a frequency-dependent complex permittivity which varies over the layers. The 100 sublayers have equal thickness (*δ* = 30 nm), each layer having a different imaginary part of the complex relative permittivity equal to $j\cdot \frac{h_{i}\sigma }{\omega \varepsilon_{0}}$, where the electrical conductivity, σ, is the sweep parameter and *h_i_* is a dimensionless weighting factor which accounts for the in-depth, exponentially degrading conductivity according to the expression hi=e−i−1δδUV·1+e−δδUV2, for *i* = 1–100 [7]. It was supposed that the UV light illumination takes place from the top or from the bottom side of the ZnO layer: in the former case, the ZnO conductivity exponentially decreases from the upper side of the stacked region, while in the latter one it decreases from the SiO_2_/ZnO interface, as shown in Figure 7.

A 2D FEM study was performed by using the Comsol 5.6 software to evaluate the resonant frequency of the Rayleigh and Sezawa waves in the unit cell configurations shown in Figure 7. A sweep parameter study was performed to calculate the phase velocity, $v=Real\left(f\right)\cdot \lambda$ (*f* being the resonant frequency), and the propagation loss, α=−54.6·ImagfRealf, of the Rayleigh and Sezawa waves at different ZnO conductivities (from 10^−3^ to 10^14^ S/m) and for different *δ*_UV_ values (from 100 to 500 nm). In the presence of variable electrical conductivity, the permittivity of the ZnO becomes complex and so do the stiffened elastic constants which are a function of the permittivity through the relation cijstiff=cij+eik·ekjεii+jσω=cij+eik·ekjεii1+σ2ω2ε02 − *j*eik·ekjσωε0εii1+σ2ω2ε02, where *c_ij_*, *e_ij_*, and ε*_ij_* are the elastic, piezoelectric, and permittivity constants; as a result, the wave velocity, which is function of the material physical constants, becomes complex [13,14]. 

**Figure 8:**
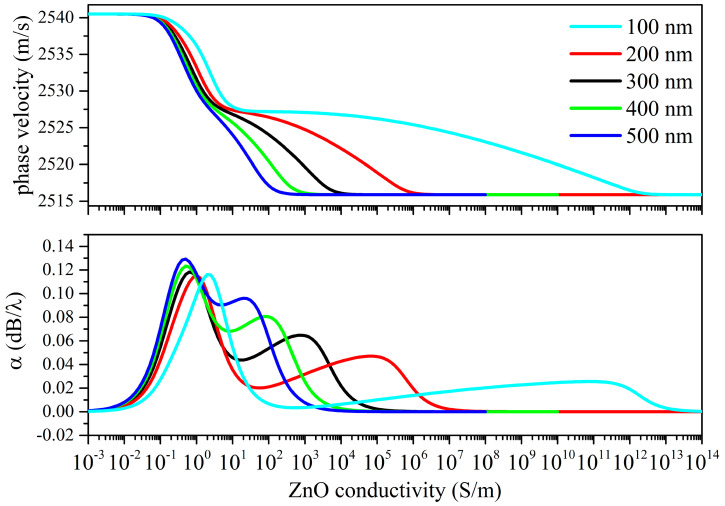
Rayleigh wave phase velocity and propagation loss vs. conductivity curves under top illumination; the penetration depth is the running parameter.

**Figure 9:**
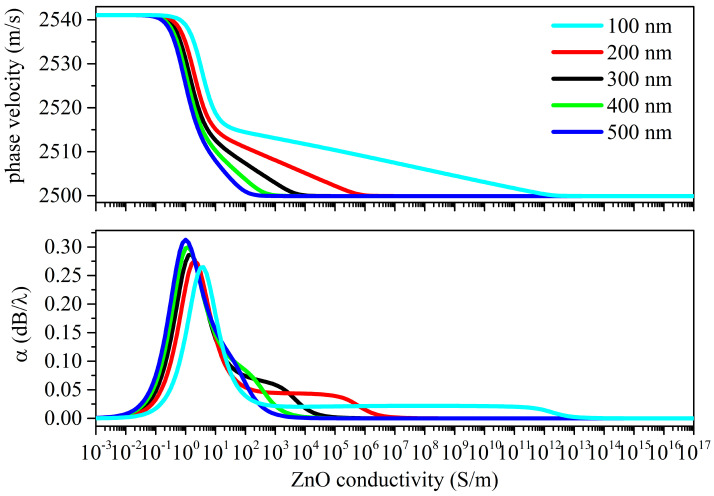
Rayleigh wave phase velocity and propagation loss vs. conductivity curves under bottom illumination; the penetration depth is the running parameter.

The *δ*_UV_ values were chosen from those reported in the available literature. In [15,16,17], a value ranging from 80 to several hundred nanometers is attributed to the UV penetration depth in ZnO at 365 nm, so different *δ*_UV_ values are strongly related to the structural properties of the photo-conducting material. Calculations of the Rayleigh wave velocity and attenuation vs. the ZnO conductivity curves were performed under both top and bottom illuminations, as shown in Figure 8 and Figure 9.

The phase velocity vs. the ZnO conductivity curves of the Rayleigh wave show two drops: the amplitude of the first velocity drop is like that of the *surface* AE effect but is moved toward abscissa values lower than those referred to as the *surface* AE effect; the second plateau becomes less pronounced for increasing *δ*_UV_ values. The magnitude of the total velocity drop exceeds that of the *surface* AE effect; the total drop resulting from the bottom illumination is larger than that of the top illumination.

The propagation loss vs. the σ curves of the Rayleigh wave under the top illumination (Figure 8) show two peaks of different amplitudes: the dominant peak is downshifted respect to the single peak of the *surface* AE effect; the second small peak moves toward the larger one with increasing *δ*_UV_ and somewhat merges to the first peak at *δ*_UV_ = 500 nm. The amplitude of the large peaks increases with increases in the *δ*_UV_.

When the first velocity plateau of the Rayleigh wave under the top illumination is reached, the propagating medium consists of a thin conductive ZnO layer on top of the lossy ZnO/piezoactive-ZnO/fused silica structure: with a further conductivity increase, the AE effect is repeated.

The thinning effect of the piezoelectric ZnO layer due to the bottom illumination (Figure 9) results in an almost linear velocity decrease just after the velocity drop; the absence of a peak in the propagation loss suggests that the wave velocity is reduced due to the mass loading effect of the lossy ZnO layer on the fused silica whose SAW velocity decreases (respective to the value of the bare fused silica substrate) as the thickness of the lossy ZnO layer increases. The propagation loss reaches the second plateau when the lossy stacked ZnO region becomes conductive.

**Figure 10:**
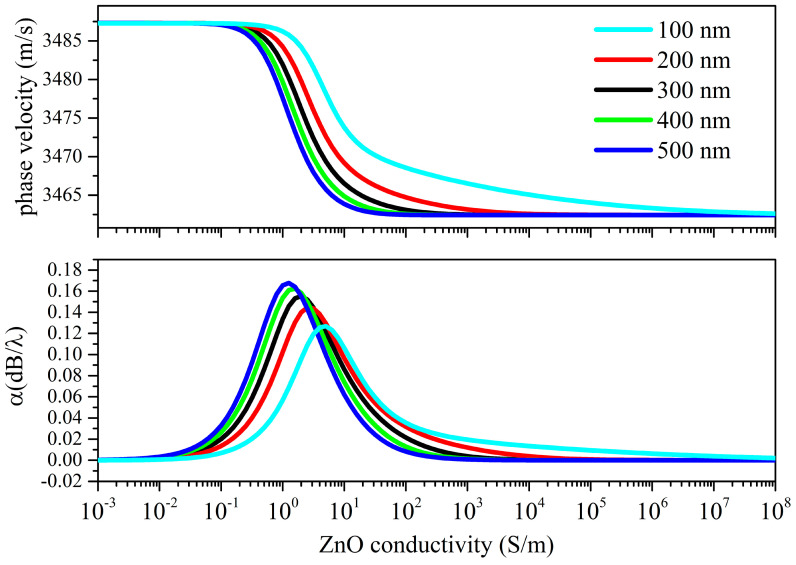
Sezawa wave phase velocity and propagation loss vs. conductivity curves under top illumination; the penetration depth is the running parameter.

**Figure 11:**
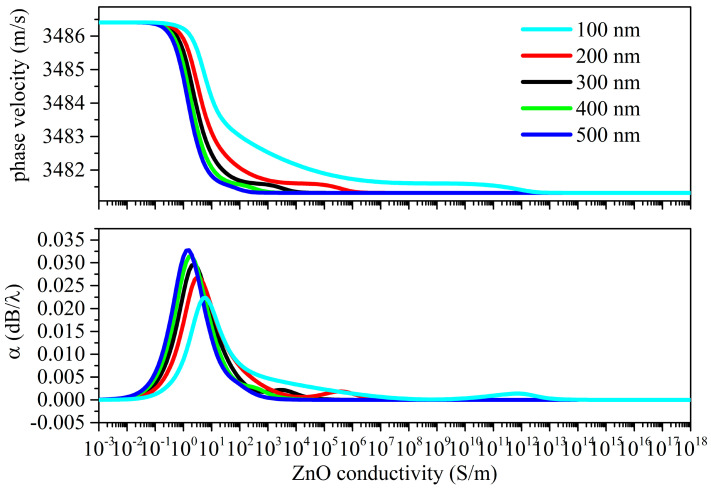
Sezawa wave phase velocity and propagation loss vs. conductivity curves under bottom illumination; the penetration depth is the running parameter.

Figure 10 and Figure 11 show the Sezawa wave velocity and attenuation vs. the ZnO conductivity curves under the top and bottom configurations.

The phase velocity vs. the ZnO conductivity curves of the Sezawa wave resulting from top illumination have a single drop which is larger than the total drop observed under bottom illumination. The electrical potential of the Sezawa wave is confined to the upper part of the ZnO layer; consequently, the propagation characteristics of the mode are more influenced by the variations in electrical conductivity caused by illumination from above than from below.

The propagation loss vs. the σ curves of the Sezawa wave under top illumination have a single peak while those under bottom illumination show a second very small peak.

## 5. Discussion

The *surface* AE response of both the Rayleigh and Sezawa waves has the same shape regardless of the type of propagating medium (whether it is a ZnO half-space or a ZnO/SiO_2_ substrate), as shown in Figure 4. The magnitude of the relative velocity drop and attenuation peak are equal to *K*^2^/2 and *K*^2^/4, as predicted by the perturbation theory: the larger the *K*^2^, the larger the expected conductometric sensitivity.

**Figure 12:**
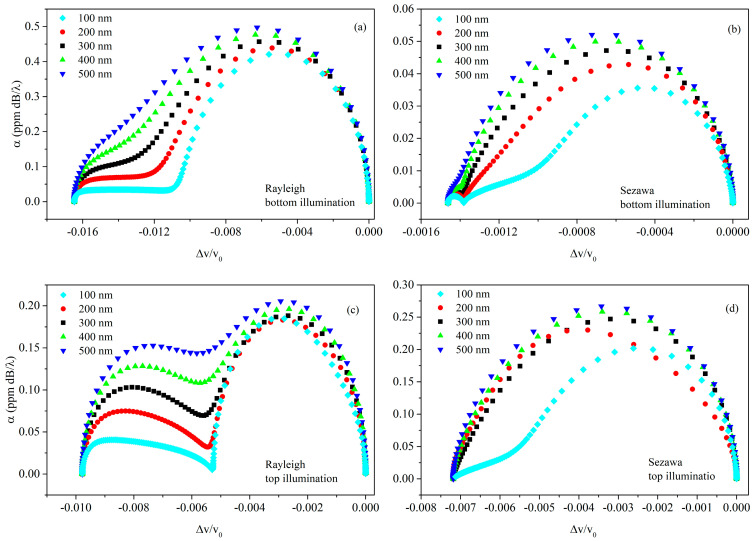
The parametric representation of the *α*/*k* vs. Δvv0 curves in ZnO/SiO_2_ for (**a**) Rayleigh wave under bottom illumination, (**b**) Sezawa wave under bottom illumination, (**c**) Rayleigh wave under top illumination, and (**d**) Sezawa wave under top illumination; σ is the variable parameter.

The *volume* AE response shows a behavior that is very different respective to the *surface* counterpart; the different behavior is particularly evident if *α*/*k* is plotted vs. ∆vv0, with σ as the variable parameter. The parametric representation of the *surface* AE response is like an elliptical arc with endpoints on the abscissa; the parametric representation of the *volume* AE response can be resolved by two semi-ellipses, as shown in Figure 12.

**Figure 13:**
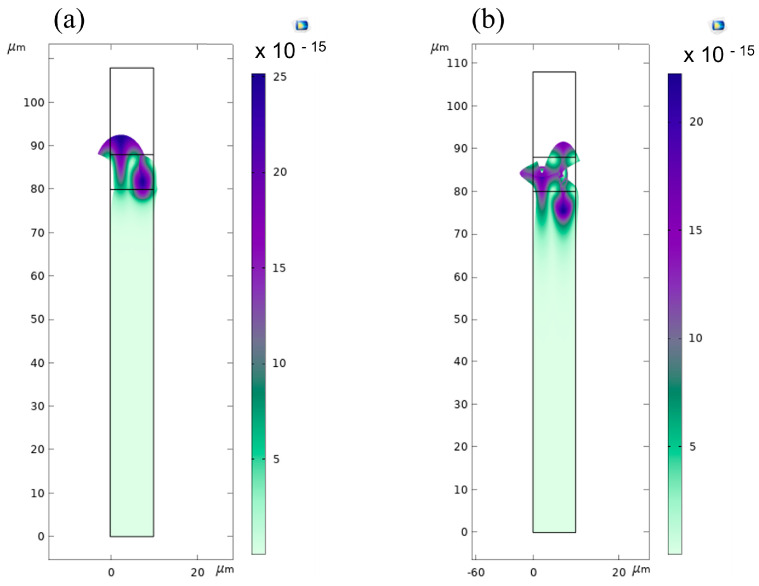
The solid displacement of the (**a**) Rayleigh and (**b**) Sezawa modes in ZnO (8 μm)/SiO_2_.

To explain the different AE behaviors of the two waves and under the two lighting configurations, it is useful to compare the mechanical displacement and the electric potential distribution in ZnO/SiO_2_. Figure 13a,b shows the solid displacement of the two waves travelling in the ZnO (8 μm)/SiO_2_ with λ = 10 μm. The displacement of the Rayleigh wave is mostly confined to nearby the air/guiding layer interface and rapidly decreases exponentially into the SiO_2_ substrate. The confinement under the Sezawa wave occurs in the layer and at the boundary between the ZnO layer and the bulk substrate.

**Figure 14:**
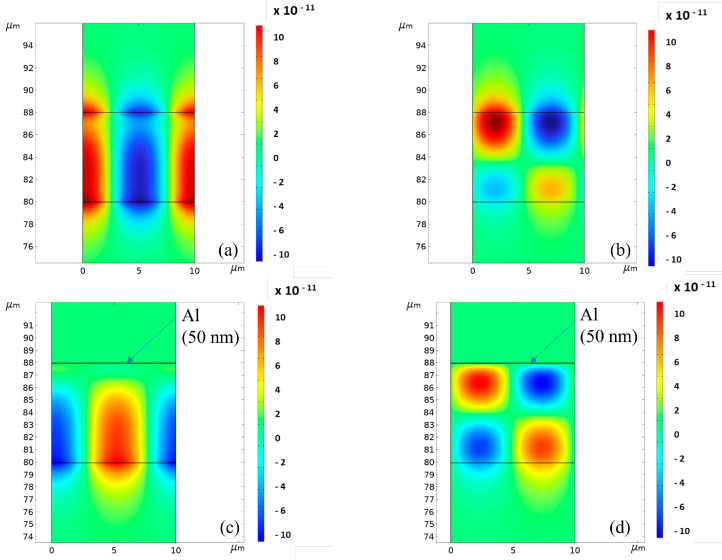
The electric potential of the (**a**) Rayleigh and (**b**) Sezawa modes in ZnO (8 μm)/SiO_2_; (**c**) that of the Rayleigh and (**d**) Sezawa waves in Al (50 nm)/ZnO (8 μm)/SiO_2_.

Figure 14a,b shows the electric potential of the Rayleigh and Sezawa waves travelling in the ZnO (8 μm)/SiO_2_ with λ = 10 μm. Φ is almost uniformly distributed inside the ZnO film under the Rayleigh wave while it is mostly confined in the upper half of the layer under the Sezawa wave. Figure 14c,d shows the electric potential of the Rayleigh and Sezawa waves travelling in Al (50 nm)/ZnO (8 μm)/SiO_2_; the Al is assumed to be electrically floating and to have a fixed permittivity. The metal layer (responsible of the *surface* AE effect) screens the *x* component of the electric field just below the free surface of the ZnO layer; as a result, this interaction feeds back into the stiffened elastic constants of the ZnO, modifying the wave velocity and attenuation.

**Figure 15:**
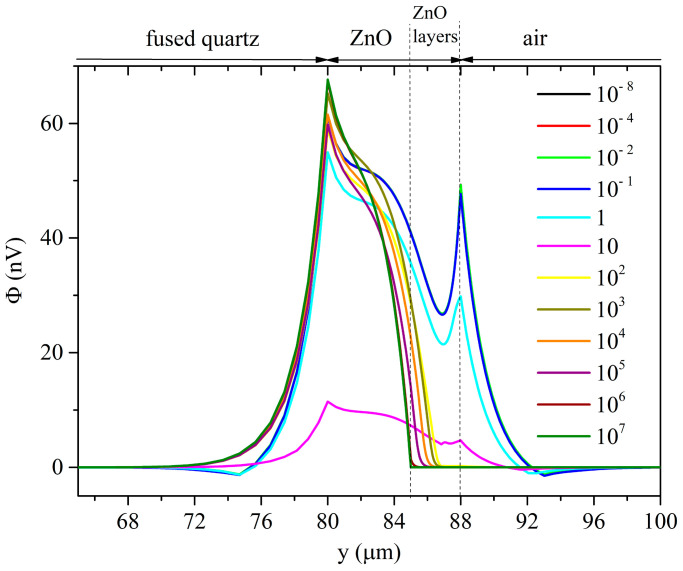
The electric potential distribution under the Rayleigh mode at some conductivity values, under top illumination, and under *δ*_UV_ = 200 nm.

**Figure 16:**
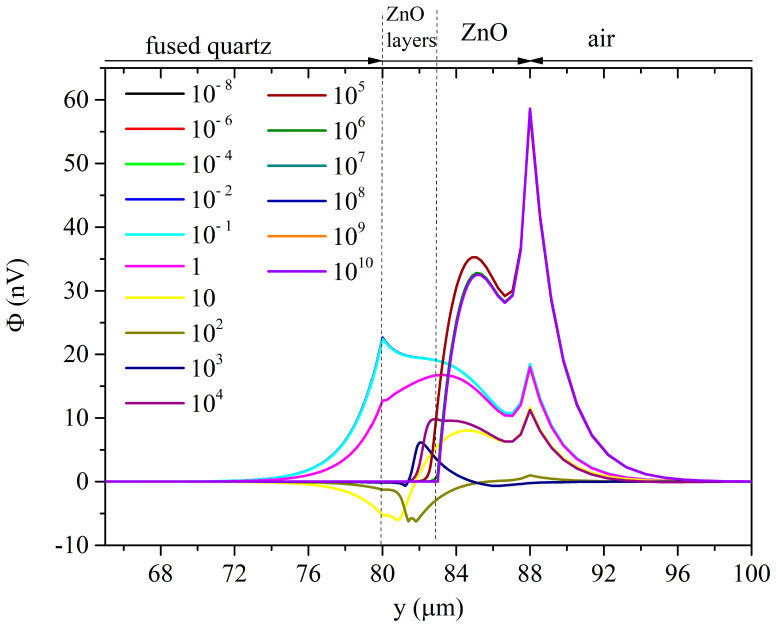
The electric potential distribution under the Rayleigh mode at some conductivity values, under bottom illumination, and when *δ*_UV_ = 200 nm.

Figure 15 and Figure 16 show, as an example, the electric potential distribution under the Rayleigh mode at some conductivity values (the running parameter), under top and bottom illumination and assuming *δ*_UV_ = 200 nm.

**Figure 17:**
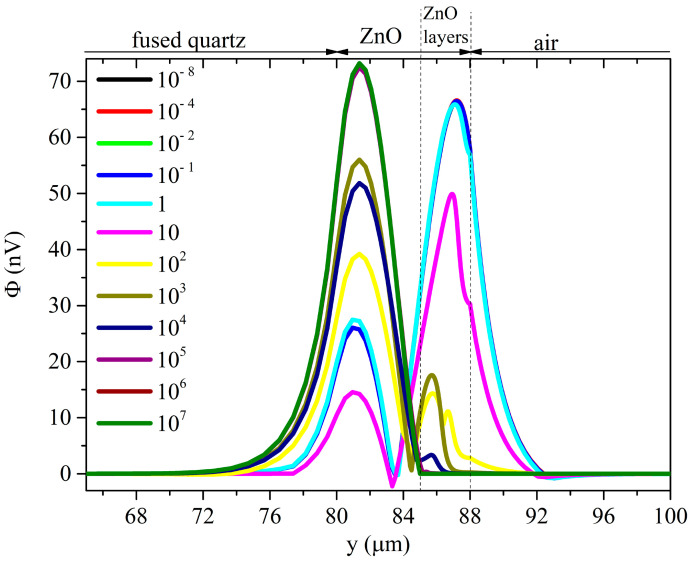
The electric potential distribution under the Sezawa mode at some conductivity values, under top illumination, and when *δ*_UV_ = 200 nm.

**Figure 18:**
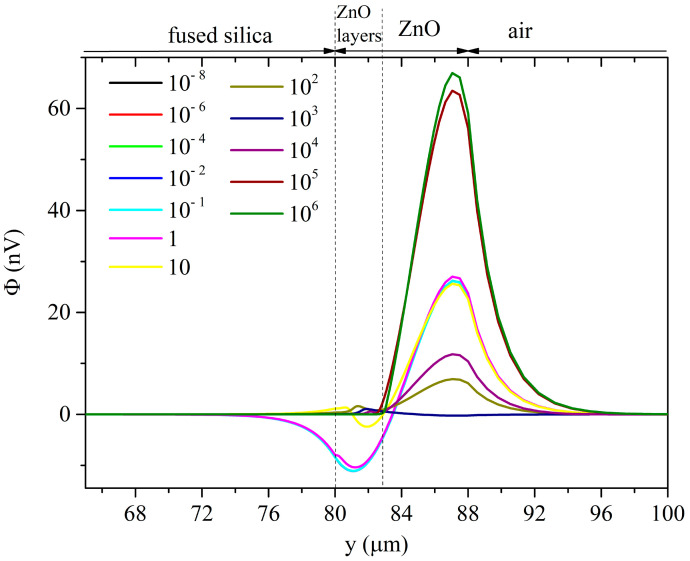
The electric potential distribution under the Sezawa mode at some conductivity values, under bottom illumination, and when *δ*_UV_ = 200 nm.

Under UV illumination, the distribution of the electrical potential within the ZnO layer is affected by a perturbation which causes the electrical conductivity to grow in magnitude and within deeper and deeper layers. Figure 17 and Figure 18 show, as examples, the electric potential distributions under the Sezawa mode at some conductivity values (the running parameter), under top and bottom illumination, and assuming *δ*_UV_ = 200 nm.

For very low conductivity values, the electric potential distribution under the Rayleigh mode is almost uniformly distributed inside the piezoelectric layer and decays exponentially in air and in fused silica. For very low conductivity values, the electric potential distribution of the Sezawa mode is mostly concentrated in the upper part of the ZnO layer and it vanishes almost in the middle of the ZnO layer.

With increasing conductivity, the potential Φ of both the two waves becomes null inside the *perturbed* stacked region; the ZnO layer is now divided into two parts—a conductive lossy layer and an unperturbed piezoelectrically active layer. Φ is short circuited in the former layer; in the latter layer, Φ satisfies the continuity boundary condition with the potential in the stacked region and in the other side (which can be air or fused silica, depending on the UV illumination type). As a result, the electric potential of the Rayleigh wave is concentrated inside the piezoelectrically active portion of the ZnO layer (not involved in the UV adsorption) where it reaches a peak at the fused silica/ZnO interface or at the ZnO/air interface, for top and bottom illumination, respectively: the magnitude of the peak under top illumination is larger than that under the bottom one. The electric potential of the Sezawa wave reaches a peak value and then it decreases just before the interface with air or with the substrate; the magnitude of the peak is larger under the bottom illumination than that under the top one.

Under the hypothesis that ZnO conductivity changes are induced by UV illumination, the resulting *volume* AE effect can be assumed as follows: 1. for low-incident UV power, the conductivity of the ZnO film is low as there are only a few charge carriers moved by the wave, so the wave velocity is almost constant and the attenuation is low; 2. for high-incident UV power, the conductivity of the film is high and the electrons in the layer experience low ohmic losses resulting in a low attenuation and constant velocity due to saturation; 3. under UV illumination, an electrically conductive ZnO (ec-ZnO) layer is formed whose thickness increases at the expense of the piezoelectrically active ZnO layer, which becomes thinner than its thickness value in the dark; 4. under UV illumination, the electrical boundary conditions and hence the *K*^2^ of the two modes changes; 5. under UV illumination, a sort of three-layer structure which is dispersive, SiO_2_/ec-ZnO/ZnO or SiO_2_/ZnO/ec-ZnO under bottom or top illumination, is formed

## 6. Conclusions

The available scientific literature reports many examples of experimental measurements of the UV sensing performances of ZnO-based SAW sensors; in Table 1, some details (the SAW device structure, the resonant frequency, the frequency shifts the device undergoes under exposure to UV power, and the corresponding references) of ZnO-based SAW UV sensors are listed.

The list is not exhaustive but it presents the general trend of the presently available research in the SAW UV sensors field: many papers show the reversibility and repeatability of the response of the ZnO-based UV sensor, and calculate its sensitivity to a single power value of the UV source; some papers measure the sensitivity of sensors based on a ZnO layer as thin as the UV penetration depth (this condition satisfies the assumption that the entire ZnO layer undergoes a homogeneous conductivity change under UV illumination); some papers investigate the sensing performances of devices based on ZnO thickness greater than the UV penetration depth but in these, the explored UV power range is not sufficiently wide to reach the saturation or to enable the double-relaxation effect. In reference [21], however, the ZnO (3.23 μm)/Si-based SAW oscillator was tested in the low power region of up to about 50 μW/cm^2^, resulting in a Sezawa wave sensitivity of 8.12 ppm/(μW/cm^2^), while a sensitivity of 1.62 ppm/(μW/cm^2^) was measured in the high-power region (from about 50 to 551 μW/cm^2^). It would have been useful to have some more measurements of both frequency shift and propagation loss over a wider range of UV powers (>551 μW/cm^2^) to verify if the hypothesis of there being an approaching plateau due to a double-relaxation phenomenon is realistic.

It is important to underline that, for a very thin ZnO layer on top of a high-*K*^2^ piezoelectric substrate, it is the latter that drives the *K*^2^ of the bilayer structure; the piezoelectric substrate is responsible for the excitation and detection of the SAW while the ZnO layer mainly acts as a UV sensing element with homogeneous conductivity distribution.

If the ZnO layer is used in combination with a non-piezoelectric substrate (such as fused silica, sapphire, diamond, or silicon, to name just a few), it recovers the double role of the UV sensitive layer and SAW transducer; in this case, the *K*^2^ dispersion curve plays a fundamental role in choosing the ZnO h/λ value which corresponds to a high *K*^2^ value. Referring to Figure 2 as well as to the *K*^2^ dispersion curves published in the literature, generally, an acceptable *K*^2^ value corresponds to a h/λ value equal to a few tens of percentage points: if a very thin (few hundreds of nanometers) ZnO thickness is chosen, it is necessary to fabricate IDTs with fingers of sub-micrometer-sized dimensions to obtain a high *K*^2^ value; this can be achieved with expensive technologies and complicated GHz band electronics. The choice of the ZnO layer thickness is driven by the need to obtain a compromise between a good electroacoustic transduction efficiency, easy fabrication technology (such as micro-meter sized IDTs), and low-cost production. The use of fused silica can be a good solution for many reasons: it is a low-cost substrate material which can be economically manufactured in large sizes; it has high radiation damage resistance and is transparent in the UV region [6]. Nevertheless, the ZnO/fused silica-based sensor device needs to be properly designed in terms of the type of UV illumination (from top or from back), type of wave (Rayleigh or Sezawa), electrical boundary condition (IDTs placed at the free surface of the piezoelectric layer or buried under it), and ZnO layer thickness.

To overcome the double-relaxation effect, thin photoconductive piezoelectric ZnO films with thicknesses smaller than the UV penetration depth are ideal to be used, as the in-depth conductivity dependence is eliminated and the ZnO conductivity can be considered homogeneous in depth. Another approach to follow is to investigate the joint dependence of the *volume* AE effect on the frequency of the modes.

To the author’s knowledge, the double-relaxation AE effect has neither been theoretically studied before with the exception of reference [7] nor verified experimentally; the present paper shows preliminary results and does not pretend to provide an exhaustive study of the *volume* AE effect, which is a complex and challenging subject for which much still needs to be further investigated in the field.

## Figures and Tables

**Table 1:** The structure of SAW UV sensors, the resonant frequency, the frequency shifts the device undergoes under exposure to UV power, and the corresponding references.

SAW UV Sensor Device	Frequency (MHz)	Frequency Shift (kHz)	UV Power (mW/cm^2^) at 365 nm	Ref.
ZnO (200 nm)/LiNbO_3_	37	170	40	[18]
ZnO (71 nm)/LiNbO_3_	35	28	0.034	[19]
ZnO (200–400 nm)/Mg:ZnO/ZnO (2 μm)/sapphire	711.3	1360	2.32	[20]
ZnO (3.23 μm)/Si	842.8	1017	0.551	[21]
ZnO (~1.5 μm)/36° Y LiTaO_3_	41.5	No significant frequency shift *	0.570	[22]
ZnO (250 nm)/128° yx LiNbO_3_	439	6000 ppm/(μW/cm^2^)	0.010 to 40	[23]
ZnO (70 nm)/silica	41.2	45	19	[24]
ZnO (500 nm)/Si	122.15 339.9	400 (3rd harmonic) 10 (fundamental)	3	[25]
ZnO (2 μm)/Corning glass 2318	211.5	70	7.6	[26]
ZnO (4–6.5 μm)/Al foil (160 μm) S0 Lamb mode	30	53.7 ppm/(mW/cm^2^)^−1^	2–25	[27]
ZnO (400 nm)/ST-quartz	196	35 50 (saturation)	<16 16–300	[28]

* at a power density of 350 Wcm^−2^, the amplitude of the Love SAW mode UV sensor decreased up to −6.4 dB with a frequency shift of ∼150 kHz under a 254 nm illumination. When this device was illuminated with 365 nm UV light at 570 Wcm^−2^, the amplitude of the transmission signal decreased only by −2.5 dB without any significant frequency shift.
